# WE’RE ALL BORN NAKED AND THE REST IS DRAG: EXPLORING DRAMA THERAPY WITH AGING DRAG ARTISTS

**DOI:** 10.1093/geroni/igae098.0747

**Published:** 2024-12-31

**Authors:** Camryn Hafner, Jahmar Ortiz

**Affiliations:** Bournewood Health Systems, Somerville, Massachusetts, United States; New York University, New York, New York, United States

## Abstract

Due to barriers of mental health stigma, limited accessibility, and ageism, older adults are severely underrepresented among those currently engaging in mental health treatment (Saunders et al., 2021). For older drag artists, additional barriers exist in the lineage of invalidation, pathologization, and discrimination which Western psychology and mental health systems have traditionally afforded gender-expansive individuals (Brewster et al., 2019). Drag artistry itself has been found to offer many salutary functions, including providing a sense of freedom and empowerment, fostering resilience through creative achievement and social support, facilitating catharsis, and/or releasing negative emotions (Knutson et al., 2018; Knutson et al., 2023). However, concerns of depression, substance abuse, stressors, and experiences of discrimination among drag artists represent continued need for inclusive, strengths-based mental health care (Knutson & Koch, 2019; Knutson et al., 2018; Knutson et al., 2023; Tillewein & Kruse-Diehr, 2021). Expressive therapies such as drama therapy and psychodrama may be well-suited to engaging with and enhancing the therapeutic potential of drag art (Nakai & Martinez Di-Carlo, 2024; Soubosky, 2021). This presentation details the findings of an exploratory study examining the coping mechanisms of drag artists (n = 5) through the lens of a drama therapy clinical assessment (Ortiz, 2024). Building upon evidence-based treatment protocols (e.g., Keisari, 2021; Lahad et al., 2013), presenters also consider best practices and implications for the treatment of aging drag artists in drama therapy.

